# Supplementary material for: Quartz-based flat-crystal resonant inelastic x-ray scattering spectrometer with sub-10 meV energy resolution
Source: Sci Rep. 2018 Jan 31;8:1958. doi: 10.1038/s41598-018-20396-z (PMC5792644; doi:10.1038/s41598-018-20396-z)
Supplement: Supplementary file 1 — Supplementary infomation [file 41598_2018_20396_MOESM1_ESM.pdf]

Quartz-based flat-crystal resonant inelastic x-ray scattering spectrometer with sub-10~meV energy resolution

Jungho Kim[1,*], D. Casa[1], Ayman Said[1], Richard Krakora[1], B. J. Kim[2,3,4], Elina Kasman[1], Xianrong Huang[1], and T. Gog[1,+]

[1]Advanced Photon Source, Argonne National Laboratory, Lemont, Illinois 60439, United States

[2]Department of Physics, Pohang University of Science and Technology, Pohang 790-784, Republic of Korea

[3]Center for Artificial Low Dimensional Electronic Systems, Institute for Basic Science (IBS), 77 Cheongam-Ro, Pohang 790-784, Republic of Korea

[4]Max Planck Institute for Solid State Research, Heisenbergstra 1, D-70569 Stuttgart, Germany

* jhkim@aps.anl.gov

+ gog@aps.anl.gov

Supplementary Figures

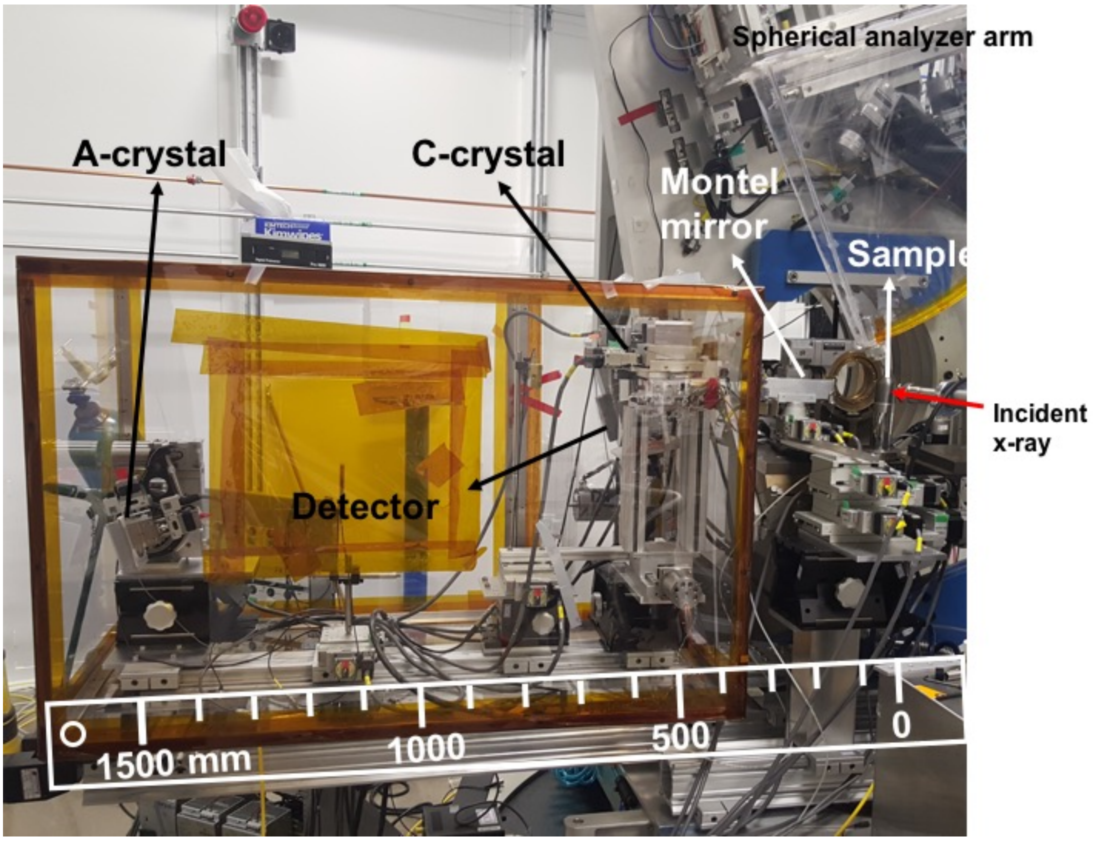

Supplementary Figure 1| Installed flat-crystal RIXS spectrometer. The flat-crystal analyzer system is installed on a 6-circle RIXS spectrometer at the sector 27-ID-B at the advanced photon source (APS). Plexiglass box is filled with a helium atmosphere to improve thermal stability and reduce air scattering and absorption. The center of the Montel mirror is located at a distance of 200 mm from the sample as designed. The center of the C-crystal is positioned at around 500 mm downstream to a collision with the Montel mirror and the existing RIXS spectrometer. The A-crystal is placed at a distance of about 1000 mm from the C-crystal to secure a proper space for the detector which is positioned below the C-cryatal and collects the reflected beam from the A-crystal. In this figure, the polarizer (P-crystal) is not shown.

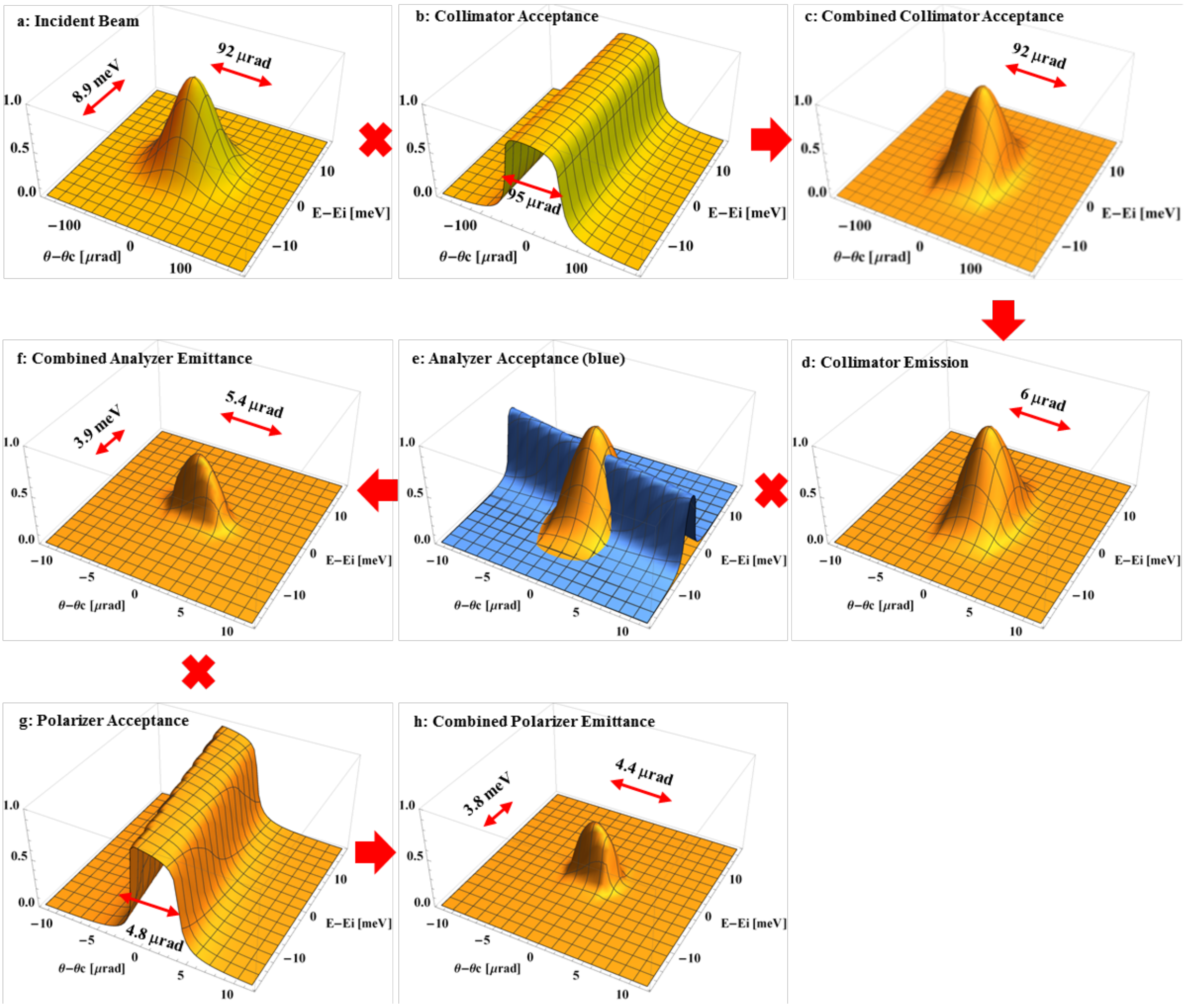

Supplementary Figure 2| Series of 3-d DuMond diagrams, showing the progression of x-rays through the analyzer system in angle-energy space. (a) The incident x-ray profile. (b) The C-crystal acceptance. (c) The x-ray profile before the C-crystal. (d) The x-ray profile after the C-crystal. (e) The A-crystal acceptance (blue). (f) The x-ray profile after the A-crystal. (g) The P-crystal acceptance. (h) The x-ray profile after the P-crystal.

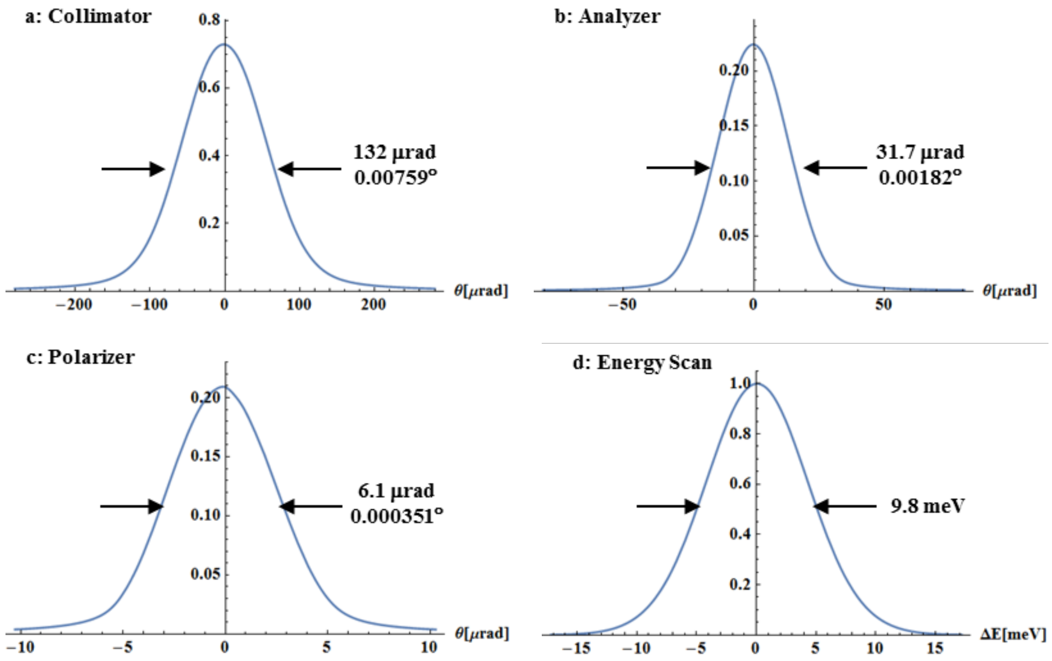

Supplementary Figure 3| Simulated rocking curves. (a) The C-crystal rocking curve scan. (b) The A-crystal rocking curve scan. (c) The P-crystal rocking curve scan. (d) The incident energy scan.

Supplementary Tables

Supplementary Table 1| Configuration of Positioning Motor Stages for the Analyzer Optical Components

Montel mirror and crystals are mounted on motorized precision stages, manufactured by Kohzu Precision Co., Ltd. These stages are equipped with 5-phase Vexta stepping motors in combination with Vexta micro-stepping motor drivers. Additionally, the analyzer crystal is installed on a 3-point kinematic mount driven by motorized Newport micrometer actuators. Some coarse motions are done with conventional 2-phase stepping motor stages. Nominal positioning resolutions are listed below. Here the linear directions adhere to a standard coordinate system, where x is horizontal and perpendicular to the x-ray beam, y is vertical and z is horizontal along the beam direction. θ is the pitch, χ is the roll and ϕ is the yaw of a component.

Component	Axis	Resolution	Component	Axis	Resolution
Montel Mirror	x	0.25 μm	Analyzer Crystal	x,y	manual
	y	0.125 μm		θ	0.62 μrad
	z	0.25 μm		χ	0.55 μrad
	θ	8.4 μrad		ϕ	0.55 μrad
	χ	10 μrad			
	ϕ	35 μrad			
Collimator Crystal	x	50 μm	Polarizer Crystal	x	0.25 μm
	y	0.125 μm		θ	0.62 μrad
	θ	1.0 μrad		χ	10 μrad
	χ	8.4 μrad			
	ϕ	35 μrad			

Supplementary Table 2| X-ray throughput. Defining "throughput" in the current context as the integrated intensity of a crystal emittance over angle and energy, as compared to the incident beam, the following progression is determined from the simulation.

Position	Supplementary Figure 2	Throughput
Incident Beam	a	1
Collimator Emittance	d	0.74
Analyzer Emittance	f	0.22
Polarizer Emittance	h	0.17

Supplementary Notes

Supplementary Note 1| Simulations

In designing the flat-crystal analyzer system, care was taken to maximize the incident solid-angle acceptance for scattered radiation emanating from the sample, while optimizing the throughput for all optical components (crystals, multilayer mirror), maintaining the best energy resolution and provide efficient polarization analysis without sacrificing any resolution. This was accomplished by carefully selecting suitable crystal reflection and asymmetry angles.

Diffraction conditions and dynamic scans of the crystal analyzer system, consisting of collimator, analyzer and polarizer, were simulated in the form of extended 3-dimensional DuMond diagrams[1], based on two-beam dynamical diffraction theory as formulated in[2]. These diagrams represent intensities of successive reflections as a function of angle and energy. Simulations were performed in the "Wolfram Mathematica 11" computing environment, using subroutines that implement pertinent dynamical diffraction formulas. An example is shown below in Supplementary Fig. 2.

Supplementary Note 2| Incident Scattered Radiation Emanating from the Multilayer Mirror

The incident radiation was modelled as a double-Gaussian, representing the energy band pass of the high-resolution monochromator along the energy axis and the emittance of the multilayer mirror along the angular axis. In the present case, two successive double-bounce, monolithic Si(844) channel-cut crystals were employed as high-resolution monochromator. The resulting energy bandpass is 8.9 meV, determined by a simulation based on dynamic diffraction theor. The multilayer mirror was designed to collimate incident scattered radiation to within 100 μrad in the vertical plane[3]. Comparing measurements with simulations, an actual emittance of 92 μrad was determined. The incident beam is shown in Supplementary Fig. 2a.

Supplementary Note 3| Collimator Crystal

For the collimator (C-crystal), a low-order, asymmetric Si reflection was chosen in order to be able to match its angular acceptance to the emittance of the preceding multilayer mirror, while maximizing the degree of collimation and the throughput. An asymmetric Si(111) crystal with an angle of 8.95° between its surface and the

diffraction planes, corresponding to an asymmetry factor of b=-0.0642, provided a well matched acceptance of 95 μrad. With the prevailing b-factor, a degree of collimation by 15.6-times was reached, resulting in an angular emission of 6 μrad. The acceptance of the C-crystal is shown in Supplementary Fig. 2b., resulting in the combined acceptance shown in Supplementary Fig. 2c, when merged with the incident beam. The asymmetric transformation leads to an emission from the C-crystal shown in Supplementary Fig. 2d, where the angular extension is now reduced 15.6-times (note the contracted angular scale in Supplementary Fig. 2c), while conditions along the energy axis are unaffected.

Supplementary Note 4| Analyzer Crystal

In order to attain the best energy-resolution, a crystal reflection at near-backscattering conditions at the incident energy associated with the Ir L3 absorption edge, 11.215 keV was required. While the more commonly used Si(844) reflection has an intrinsic angular width of 17.4 μrad, corresponding to and intrinsic energy width of 14.6 meV, the availability of near-ideal crystals of α-quartz allowed for a choice of even higher resolution. Quartz(309) has an intrinsic angular width of 11.5 μrad, however, at a Bragg angle of 88.6º, much closer to back-scattering than Si(844), this angular width corresponds to an intrinsic energy width of only 3.7 meV. Moreover, the angular acceptance is more than adequate to accommodate the full beam from the collimator, thus, a symmetric quartz(309) crystal was implemented as the analyzer (A-crystal). Supplementary Fig. 2e shows the acceptance of the A-crystal in blue, crossing the emission from the collimator and resulting in the A-crystal emission shown in Supplementary Fig. 2f with an intrinsic energy width of 3.9 meV.

Supplementary Note 5| Polarizer Crystal

For the polarizer (P-crystal) to function it needs to have a Bragg angle of close to 45º. At an energy of 11.215 keV, Si(444) has a Bragg angle of 44.8 º, and is therefore suitable as a polarizer. However, with an angular acceptance of the symmetric Si(444) reflection of only 4.8 μrad, an asymmetric crystal with a b-factor of at least -0.6 is desirable to capture the full beam from the analyzer. Nevertheless, due to availability reason, a symmetric Si(844) crystal was used as a polarizer in the present initial

implementation of the spectrometer. The polarizer acceptance is shown in Supplementary Fig. 2g and the resultant emittance is shown in Supplementary Fig. 2h.

Supplementary Note 6| Rocking Curve and Incident Energy Scans.

Rocking curve scans are a useful tool for judging the quality and strain-free mount of a crystal. In simulating rocking curve scans, the acceptance of the crystal in question is translated along the angular axis, traversing the emittance of the preceding optical system. For every point a convolution of acceptance and emittance is performed. Supplementary Fig. 3a, 3b, and 3c show angular rocking curve scans obtained for the C-crystal, A-crystal, and P-crystal polarizer, respectively. For an incident energy scan in Supplementary Fig. 3d , the incident beam profile is translated along the energy axis, keeping the acceptance of the Montel mirror and successive reflectivities static.

Supplementary References

1. DuMond, J. W. Theory of the use of more than two successive x-ray crystal reflections to obtain resolving power. *Phys. Rev.* 52, 872 (1937).

2. Authier, A. *Dynamical Theory of X-Ray Diffraction (International Union of Crystallography Monographs on Crystallogra- phy, 11)* (Oxford University Press, 2001).

3. Kim, J. *et al.* Collimating montel mirror as part of a multi-crystal analyzer system for resonant inelastic x-ray scattering. *J. Synchrotron Rad.* 23, 880–886 (2016).
